# The nuts and bolts of conducting an integrated process evaluation: a presentation on the role and methods of the process evaluation integrated into a trial of a complex educational intervention for young people with diabetes (CASCADE)

**DOI:** 10.1186/1745-6215-14-S1-O53

**Published:** 2013-11-29

**Authors:** Mary Sawtell, Meg Wiggins

**Affiliations:** 1 Institute of Education, London, UK

## 

The CASCADE study was a pragmatic cluster RCT, with integral process and economic evaluations, of a complex psycho-educational intervention for young people with diabetes. The study was funded by the NIHR-HTA and carried out by a multi-institutional team (UCL, IoE, LSHTM, & School of Pharmacy).

The extensive integrated multi-method process evaluation was planned prospectively and ran for the four years of the trial. The aims of the process evaluation were to 1) assess the feasibility and describe the provision of the CASCADE intervention within a standard clinic setting for a diverse range of young people; and 2) build on and help explain trial outcome findings and provide information on how the intervention might be modified.

The process evaluation used a range of both qualitative and quantitative methods including observations of education sessions; interviews with a sub sample of young people, parents and staff; questionnaires relating to views of the intervention; attendance data and case note review.

Currently there is limited published guidance on how to conduct a high quality integrated process evaluation that improves the usefulness of trial findings. In this presentation, members of the CASCADE process evaluation research team will provide a detailed example of carrying out such an evaluation. The presentation will include; the justifications for the methodological approach used, descriptions of context and sampling, data collection and analysis methods. Attention will be drawn to the specific methodological processes and practical facilitators employed to maximize the integration of trial and process components.

